# Alterations in the transcriptome and antibiotic susceptibility of *Staphylococcus aureus *grown in the presence of diclofenac

**DOI:** 10.1186/1476-0711-10-30

**Published:** 2011-07-21

**Authors:** James T Riordan, JoAnne M Dupre, Stephanie A Cantore-Matyi, Atul Kumar-Singh, Yang Song, Shahrear Zaman, Sonia Horan, Nada S Helal, Vijayaraj Nagarajan, Mohamed O Elasri, Brian J Wilkinson, John E Gustafson

**Affiliations:** 1 Department of Cell Biology, Microbiology and Molecular Biology, University of South Florida, Tampa, FL 33620, USA; 2 Microbiology Group, Department of Biology and Molecular Biology Program, New Mexico State University, Las Cruces, NM 88003, USA; 3 Department of Biology, Illinois State University, Normal, IL 61790, USA; 4 Department of Biological Sciences, University of Southern Mississippi, Hattiesburg, MS 39406, USA; 5 Bioinformatics and Computational Biosciences Branch (BCBB), OCICB/OSMO/OD/NIAID/NIH, Bethesda, MD 20892, USA

**Keywords:** Diclofenac, *S. aureus*, antibiotic resistance, non-steroidal anti-inflammatory drugs (NSAIDs)

## Abstract

**Background:**

Diclofenac is a non-steroidal anti-inflammatory drug (NSAID) which has been shown to increase the susceptibility of various bacteria to antimicrobials and demonstrated to have broad antimicrobial activity. This study describes transcriptome alterations in *S. aureus *strain COL grown with diclofenac and characterizes the effects of this NSAID on antibiotic susceptibility in laboratory, clinical and diclofenac reduced-susceptibility (Dc^RS^) *S. aureus *strains.

**Methods:**

Transcriptional alterations in response to growth with diclofenac were measured using *S. aureus *gene expression microarrays and quantitative real-time PCR. Antimicrobial susceptibility was determined by agar diffusion MICs and gradient plate analysis. Ciprofloxacin accumulation was measured by fluorescence spectrophotometry.

**Results:**

Growth of *S. aureus *strain COL with 80 μg/ml (0.2 × MIC) of diclofenac resulted in the significant alteration by ≥2-fold of 458 genes. These represented genes encoding proteins for transport and binding, protein and DNA synthesis, and the cell envelope. Notable alterations included the strong down-regulation of antimicrobial efflux pumps including *mepRAB *and a putative *emrAB/qacA*-family pump. Diclofenac up-regulated *sigB *(σ^B^), encoding an alternative sigma factor which has been shown to be important for antimicrobial resistance. *Staphylococcus aureus *microarray metadatabase (SAMMD) analysis further revealed that 46% of genes differentially-expressed with diclofenac are also σ^B^-regulated. Diclofenac altered *S. aureus *susceptibility to multiple antibiotics in a strain-dependent manner. Susceptibility increased for ciprofloxacin, ofloxacin and norfloxacin, decreased for oxacillin and vancomycin, and did not change for tetracycline or chloramphenicol. Mutation to Dc^RS ^did not affect susceptibility to the above antibiotics. Reduced ciprofloxacin MICs with diclofenac in strain BB255, were not associated with increased drug accumulation.

**Conclusions:**

The results of this study suggest that diclofenac influences antibiotic susceptibility in *S. aureus*, in part, by altering the expression of regulatory and structural genes associated with cell wall biosynthesis/turnover and transport.

## Background

*Staphylococcus aureus *is a human pathogen associated with integumental infections and life-threatening systemic diseases, such as sepsis and endocarditis. The tendency of *S. aureus *to acquire antibiotic resistance has led to the global dissemination of clones expressing multiple antimicrobial resistance including some that express intermediate or full resistance to the glycopeptide vancomycin [1-3]. Intrinsic mechanisms of antibiotic resistance (i.e. those not acquired by mutation or lateral genetic transfer) in *S. aureus*, might facilitate the acquisition of clinical resistance by allowing for protracted survival in the presence of subinhibitory drug concentrations [4,5]. This could, in part, be achieved by reducing the intracellular concentration of antibiotics due to the up-regulation of drug efflux systems and alterations in membrane permeability [6]. Intrinsic resistance mechanisms can be induced upon exposure to antibiotics, as well as chemical repellants, such as the non-steroidal anti-inflammatory drug (NSAID) salicylate [7]. Salicylate, the principal pharmacoactive metabolite of aspirin, has been shown to induce reduced susceptibility to mechanistically-unrelated antimicrobials by both efflux-dependent and -independent mechanisms in *S. aureus *[8-12], and in various Gram-negative bacteria [7]. Salicylates have also been shown to inhibit growth of staphylococci at therapeutically-relevant concentrations [13-15].

The NSAID diclofenac is antibacterial *in vitro*, and administration to mice or rats infected with *Listeria monocytogenes, Salmonella typhimurium, Mycobacterium tuberculosis *or *S. aureus *has been reported to significantly reduce bacterial pathogen cell counts in blood and in organ homogenates [16-18]. Growth of *E. coli *with inhibitory concentrations (2 × MIC or 100 μg/ml) of diclofenac was shown to reduce the rate of Ci (^3^H) deoxythymidine incorporation into DNA, indicating that diclofenac may target DNA biosynthesis [19]. As for salicylate and other NSAIDs, diclofenac probably acts on multiple targets in the cell. For example, the antibacterial effects of salicylate have been attributed to the down-regulation of adhesins and toxin production [20,21], the alteration of central and energy metabolism [8,22,23], and physiochemical effects on internal pH and membrane potential [24].

Diclofenac has been shown to increase the susceptibility of bacteria *in vitro *to streptomycin and to act synergistically with streptomycin, other aminoglycosides, and cephalosporins to reduce bacterial pathogen counts in animals [25-27]. This could result from any combination of diclofenac-inducible host- or bacteria-specific effects, or through chemical interactions between diclofenac and antibiotics. For example, diclofenac stimulates pro-inflammatory cytokines such as TNF-α and IFN-γ in BALB/c mice [28], and has been observed to improve the pharmacokinetic properties of ceftriaxone and cefotiam in a rabbit model of experimental *E. coli *endocarditis [26]. Diclofenac may also alter the expression of bacterial antibiotic resistance genes, as has been shown for salicylate [7]. Salicylate is a ligand for transcriptional regulators of multidrug resistance, such as the multiple antibiotic resistance regulator (MarR) protein of *E. coli *[29], and alters the expression of MarR-family genes such as *sarA, sarR*, and *mgrA *in *S. aureus *[8,9].

The effect of diclofenac on antimicrobial resistance has thus far been determined for drugs which have limited therapeutic value for *S. aureus *infections. This includes the psychotropic drug trifluoperazine [30], and the aminoglycoside, streptomycin [25]. In addition, the changes in bacterial gene expression which occur in response to diclofenac have not been reported. The present study describes transcriptome alterations in the methicillin-resistant *S. aureus *(MRSA) strain COL when grown with diclofenac. Furthermore, the effect of diclofenac on the susceptibility of laboratory, and antibiotic-resistant clinical strains to several classes of antibiotics was determined.

## Methods

### Strains, chemicals and growth conditions

For a complete list of *S. aureus *strains used in this study see Table 1. Strains were stocked in glycerol (20% vol/vol) at -80°C. Working cultures were grown on Mueller Hinton agar (MHA) or tryptic soy agar (TSA) and maintained at 4°C. Overnight cultures (18 h, 37°C, 200 RPM) were prepared by inoculating single colonies into MHB, TSB or Luria Bertani broth (LB). All NSAIDs and antibiotics were purchased from Sigma Chemical Co. (St. Louis, MO), except when indicated. Stocks of ciprofloxacin (kind gift of Bayer Corporation, West Haven CT), ofloxacin, oxacillin, and vancomycin were prepared in double-distilled water, and stocks of chloramphenicol, norfloxacin, and tetracycline were prepared in 100% ethanol. Antibiotic stock solutions (25 mg/ml) were filter-sterilized (0.2 μm) and stored at -20°C. NSAID stock solutions of acetaminophen (0.5 M), acetylsalicylic acid (0.5 M) and ibuprofen (0.4 M) were made-up in 100% ethanol; sodium diclofenac (0.15 M) was made up in methanol, and sodium benzoate (1 M) and sodium salicylate (0.5 M) stocks were prepared in distilled water. The effect of diclofenac on growth in TSB was measured for SH1000, COL and diclofenac reduced-susceptibility (Dc^RS^) mutants by measuring optical density at 580 nm (OD_580_) every hour for 8 h. For transcriptional analysis, fresh TSB cultures of strain COL were prepared by inoculating at 1:100 (vol/vol) from overnight TSB cultures. Cultures (biological replicates: N = 4 arrays; N = 3 qRT-PCR) were then grown to exponential phase (OD_580 _= 0.5) before the addition of diclofenac (80 μg/ml final concentration), or an equal volume of sterile methanol (0.16% vol/vol) for microarrays or sterile water for qRT-PCR as controls, and incubated for an additional 15 min before sampling. There was no significant difference in the expression patterns of genes between controls (see results for qRT-PCR validation of microarray genes).

**Table 1:** Strains used in this study

Strain name	Relevant strain characteristics	Reference
SH1000	Derivative of 8325-4, *rsbU+*	[85]
SC1	Derivative of SH1000, Dc^RS^	This study
COL	*mec+*, Oxa^R^	[86]
SC4	Derivative of COL, Dc^RS^, Oxa^R^	This study
BB255	Derivative of NCTC 8325, *rsbU*	[87]
WBG8287	Clinical isolate, *mec+*, Oxa^R^	[12]
WBG9312	Clinical isolate, Cip^R^	[12]
SA1199B	Cip^R^	This study

### RNA purification and cDNA synthesis

Purification of RNA and the synthesis of cDNA for microarrays and quantitative real-time PCR (qRT-PCR) followed previously described methods [8,31]. Briefly, samples were added to RNA Protect (Qiagen, Valencia, CA) and processed according to the manufacturer's instructions. Cells were harvested by centrifugation (8,000 × *g*, 20 min, 4°C) and then resuspended in 1 ml Trizol (Invitrogen, Carlsbad, CA) and processed in an FP120 FastPrep cell disruptor (MP Biomedicals, Irvine, CA). Chloroform was subsequently added to the lysates, followed by centrifugation (16,000 × *g*, 15 min, 4°C) and RNA was precipitated 1:1 (vol/vol) in 100% ethyl alcohol. The RNA was then purified using the RNeasy™ kit (Qiagen) according to the manufacturer's instructions. Contaminating DNA was removed from purified RNA using DNA*free *(Ambion, Austin, TX). For microarrays, cDNA was produced using SuperScript II Reverse Transcriptase (Invitrogen) from 2 μg of total RNA combined with random hexamers, 0.25 mM deoxynucleoside triphosphate, and 0.25 mM aminoallyl-dUTP. For qRT-PCR, cDNA was prepared as above with the exclusion of aminoallyl-dUTP.

### *S. aureus *DNA microarray hybridization and analysis

Hybridization of synthesized cDNAs to *S. aureus *DNA microarrays TIGR slides ver. 6 (http://pfgrc.jcvi.org/index.php/microarray/array_description/staphylococcus_aureus/version6.html) followed previously described protocols [8,31]. Hybridized arrays were scanned with a GenePix 4000B Microarray Scanner (Axon Instruments, Union City, CA) and LOWESS normalized TIFF images were analyzed using Spotfinder ver. 3.2.1 (JCVI). Statistical analysis was performed using a Significance Analysis of Microarrays (SAM) [32] unpaired contrast, available through the TM4 software package (JCVI). A false discovery rate of 0.05 and at least a 2-fold upregulation or downregulation in expression levels was used to assign a critical cutoff for significance. Microarray data was also compared to published *S. aureus *gene expression microarray datasets using the *Staphylococcus aureus *Microarray Metadatabase (SAMMD) as described [33]. Microarray intensity data files have been deposited in NCBI Gene Expression Omnibus (series accession number GSE30724) (http://www.ncbi.nlm.nih.gov/geo/).

### Quantitative real-time PCR

Quantitative real-time PCR (qRT-PCR) was used to validate microarray data as described [8]. Control (uninduced) and diclofenac-induced cDNAs were used in qRT-PCR with an iCycler iQ Real-Time PCR Detection System (Bio-Rad, Hercules, CA) and SYBR Green Supermix (Bio-Rad). Gene-specific primers are listed in Additional File 1. Critical threshold values were normalized using the 23S rRNA gene *rrlA *and the average (N = 3 biological replicates; N = 2 technical replicates) relative change in gene expression was reported using the method of Pfaffl [34].

### Agar diffusion MICs, and the gradient plate technique

For agar diffusion minimum-inhibitory concentration (MIC) determination, overnight *S. aureus *MHB cultures were diluted to an OD_625 nm _= 0.01 in fresh MHB. Two microliters of each diluted culture was then plated onto MHA plates containing increasing concentrations of antibiotic with 0 μg/ml (control), 32 μg/ml or 64 μg/ml of diclofenac, or diclofenac alone (control). Plates were allowed to air-dry (approx. 15 min), and were then inverted and incubated at 37°C for 24 h. The MIC was determined as the lowest concentration of antibiotic (with and without diclofenac) at which there was no visible growth. Gradient plates were utilized to determine the effect of diclofenac on antibiotic and NSAID susceptibility as described [35]. Differences in average (N = 3) MICs or distance (mm) grown into gradient plates were analyzed statistically by analysis of variance.

### Ciprofloxacin accumulation assay

Ciprofloxacin accumulation assays were performed using a Hitachi F2000 Fluorescent Spectrophotometer (Hitachi High Technologies America, Inc., Schaumburg, Ill) as described [10,36], and using exponential (OD_580 _= 0.5) cultures of strain BB255 grown in LB (control) or LB containing 32 μg/ml diclofenac. Differences in ciprofloxacin accumulation (ng antibiotic/mg dry cell weight) were analyzed using a Student's *t*-test, N = 6.

## Results

### The transcriptome of *S. aureus *grown in the presence of diclofenac

Gene expression microarray analysis was used to measure transcriptome alterations in response to growth in the presence of a subinhibitory concentration of diclofenac. The addition of 80 μg/ml diclofenac to exponential cultures of *S. aureus *strain COL resulted in the significant alteration in expression by ≥2-fold of 458 genes, representing 16.8% (458/2723) of COL genome ORFs (GenBank:CP000046); 226 of which were up-regulated, and 232 down-regulated (Additional File 2). The prevailing ontology of altered genes included those involved in transport and binding (61/459), protein synthesis (32/459) and the cell envelope (24/459). In addition, genes encoding hypothetical proteins represented 33.1% (152/459) of those significantly altered (Additional File 3).

Genes involved with resistance to antibiotics, disinfectants, and antimicrobial peptides were altered during growth with diclofenac. Many of these were down-regulated. For example, *mepR*, encoding a multiple antibiotic resistance regulator (MarR)-family protein was down- regulated -2.8-fold. MarR is a transcriptional repressor of the *marRAB *operon in *E. coli*. The expression of *marRAB *is important for *E. coli *multidrug resistance, and has been shown to be induced by salicylate [27,29,37]. Kaatz *et al*. [38] reported an increase in expression of *mepR *in multidrug-resistant *S. aureus*, in addition to two genes directly downstream and contiguous with *mepR*, which together constitute the *mepRAB *operon. The *mepA *gene encodes a multidrug and toxin family extrusion (MATE) efflux pump, and *mepB *encodes a hypothetical protein of unknown function. MepRAB confers reduced susceptibility to fluoroquinolones, tigecycline, and various biocides [39,40]. Importantly, diclofenac induction also led to the down-regulation of *mepA *(-9.2-fold) and *mepB *(-2.8-fold), revealing that the *mepRAB *operon is being repressed in its presence.

Growth with diclofenac also led to the down-regulation (-24.2-fold) of a TetR-family regulator, SACOL2593. TetR-family proteins are broadly distributed among bacteria, and have been shown to reduce expression of antimicrobial resistance through negative regulation of drug transporters [41]. For example, the *S. aureus *TetR regulator QacR represses transcription of *qacA*, encoding a major facilitator superfamily (MFS) drug transporter important for resistance to antiseptics [42,43]. TetR-family proteins also control genes involved in metabolism and in adaptation to changing environments or stressors [41]. SACOL2593 shares only 14% amino acid identity with QacR, and is similarly limited in homology with other characterized TetR-family regulators, but it is conserved among sequenced *S. aureus *strains in GenBank.

Four genes encoding putative MFS drug transporters were altered in response to diclofenac. Only one of these, SACOL0086, was up-regulated (3-fold) and its function is unknown. SACOL0086 shares 69% amino acid identity with the putative EmrB/QacA drug transporter SACOL1475, and 59% and 36% identity with the MFS transporters SACOL2449 and SACOL026, respectively. Down-regulated MFS transporters included SACOL2347 (-12.8-fold) and SACOL2348 (-40.7-fold), encoding an EmrB/QacA- and an EmrA-family drug efflux system, respectively. The *E. coli *multidrug efflux system (*emrRAB*) confers resistance to various antimicrobials, including quinolone antibiotics [44,45]. EmrR is a MarR-family repressor of *emrAB*, and like *marRAB*, the *emr *operon is inducible by salicylate [45]. Interestingly, Delgado *et al*. [31] observed a 17-fold up-regulation of SACOL2347 in the presence of fusidic acid, indicating that the expression of this putative efflux system is sensitive to both NSAIDs and antibiotics. Immediately downstream of SACOL2347-2348 is the divergently-transcribed gene SACOL2349, which encodes a conserved but uncharacterized TetR/AraC-family regulator; this gene was not, however, significantly altered in expression. Also down-regulated was the uncharacterized MFS drug transporter, SACOL2159 (-2-fold), and a multiple resistance and pH adaptation (MRP)-type transporter SACOL2156 (-2.2-fold).

Several cell envelope genes linked to antibiotic resistance were altered in response to diclofenac. This included the down-regulation of penicillin-binding protein genes *pbpB *(-3-fold) and *pbp4 *(-2.3-fold), which are involved in peptidoglycan biosynthesis and cell growth. Mutations which inactivate *pbp4 *have been identified in vancomycin resistant strains selected in the laboratory [46]. In addition, the *dlt *operon genes *dltAB*, encoding proteins involved in D-alanine metabolism were also down-regulated. Mutations in this operon have been shown to increase the sensitivity of *S. aureus *to antimicrobial peptides [47].

Diclofenac induction was observed to up-regulate *sigB *(2-fold) encoding σ^B^, an alternative sigma factor which directs the transcription of more than one hundred genes in response to stressors [48,49]. An intact *sigB *has been determined to be important for intrinsic antimicrobial resistance in *S. aureus *[35], and *sigB *is up-regulated by salicylate [9]. Diclofenac was also found to up-regulate *rsbW *by 2.3-fold. This gene encodes an anti-σ^B ^protein that sequesters cytosolic σ^B ^and interferes with its ability to associate with RNA polymerase [50]. σ^B ^is largely regulated at the post-translational level, and induction of σ^B ^upon exposure to stress is through the phosphatase activity of RsbV on RsbW, which results in the dissociation of σ^B ^and RsbW [51]. Thus alterations in *sigB *transcript levels may not correlate with altered σ^B ^activity. However, in support of σ^B ^up-regulation, comparison of diclofenac-induced microarray data with publicly available microarray datasets using SAMMD [33] revealed that 46% of the genes which are regulated by σ^B ^are also altered in expression upon exposure to diclofenac. This included a 6-fold increase in *asp23*, encoding alkaline shock protein, and shown to be an indicator of σ^B^-directed transcription [50,52,53].

Genes encoding virulence-associated proteins were significantly altered by diclofenac. For example, the staphylococcal respiratory response genes *srrA *and *srrB *were up-regulated 4.9- and 3.1-fold, respectively. When overexpressed, *srrAB *down-regulates virulence factors such as *agr *RNAIII, *tsst-1 *and *spa*, and leads to a reduced virulence in a rabbit model of endocarditis [54-56]. The *srrAB *system is also up-regulated under conditions of anaerobic growth [57]. The sensory histidine kinase gene *saeS *was down-regulated -2.8-fold in the presence of diclofenac. Rogasch *et al*. [58] have shown that the loss of *saeS *and the response regulator *saeR*, results in reduced expression of extracellular and cell surface-associated virulence factors. In agreement with *saeS *down-regulation, *cap *genes encoding capsular polysaccharide serotype 5 (CP5) were shown to be up-regulated by diclofenac; an *saeS *mutant demonstrates increased *cap *gene expression and CP5 production [59]. Down-regulated CP5 genes included those involved in chain-length determination (*cap5A *and *cap5B*) by -20.1- and -8.3-fold, as well as O-acetylation (*cap5H*) by -3.3-fold, respectively. Importantly, CP5 is one of the most prevalent *S. aureus *capsule serotypes among human clinical isolates [60], and strains null for CP5 production are more susceptible to phagocytosis, and are less virulent in a model of murine bacteremia [61-63].

Genes involved in central and energy metabolism, as well as in the metabolism of amino and fatty acids, DNA, and metabolic cofactors accounted for >30% of those significantly altered in response to diclofenac. This included the up-regulation of genes important for anaerobic growth, such as *srrAB *(above). In addition, the nitrate/nitrite respiration genes nitrate reductase (*narG*) and nitrite reductase (*nirB*) were strongly up-regulated 12.1- and 20.4-fold, and the nitrite transporter, *narK *was upregulated 31-fold, respectively. Nitrate can be used by staphylococci as an alternative electron acceptor to drive oxidative phosphorylation, reducing nitrate to nitrite via nitrate reductase A (NarGHI) [64,65]. Nitrite can then be extruded from the cell via NarK, or it can be further reduced to ammonia by NirB. Nitrate reduction can also be coupled to the fermentation of organic acids such as formate to allow for survival in the presence of stressors which dissipate proton-motive force (PMF) [66,67]. Importantly, NSAIDs such as salicylate have been shown to uncouple oxidative phosphorylation and deplete PMF in mitochondria (reviewed in [68]). In support of organic acid fermentation in the presence of diclofenac, both formate (SACOL0301) and lactate (SACOL2363) transporters were strongly up-regulated 16.1- and 25.9-fold. Finally, genes of the urease operon (*ureABCEF *and *ureD*) were shown to be down-regulated (-3.5- to -11-fold) by diclofenac. These genes encode the urease enzyme (UreABC) or are accessory to its formation, and catalyze the conversion of urea to ammonia and carbon dioxide.

Diclofenac altered the expression of genes involved in DNA stability and repair. This included the down-regulation of *radA*, SACOL1154, *recU, topA, parC, xerD *and *nfo *(-2.0- to -3.7-fold). These encode a DNA repair protein, a DNA strand exchange inhibitor, an endonuclease, topoisomerase I and the A subunit of topoisomerase IV, a tyrosine recombinase, and endonuclease IV, respectively. Up-regulated DNA repair genes included *lexA *(2.6-fold), *hexA *(2-fold), SACOL0751 (2.6-fold), encoding the repressor of the global SOS DNA repair system, a mismatch-repair protein, and a putative photolyase, respectively. Genes of the pyrimidine DNA biosynthesis *pyr *operon were also strongly down-regulated (2.9- to 14.2-fold). This finding is concordant with a previous study demonstrating impaired DNA biosynthesis in response to growth of *E. coli *with diclofenac [19].

### Quantitative real-time PCR (qRT-PCR) validation of microarray genes

Ten genes which were altered in expression as determined by microarray analysis were validated using qRT-PCR. This included genes with roles in antimicrobial resistance (*mepR, mepA*, SACOL2347), virulence (*cap5A, srrA, sigB*) metabolism (*nirB*, SACOL0301) and with other functions. The expression ratios of these genes were shown to be in strong agreement by correlation analysis (r^2 ^= 0.92) between both approaches (Additional File 2).

### Diclofenac induced alterations in susceptibility to antibiotics

Diclofenac down-regulated structural and regulatory genes of drug transport systems and other mechanisms, which may lead to alterations in phenotypic resistance to antimicrobials. To examine this possibility, the susceptibility of lab and clinical strains to seven antibiotics was examined by determining agar diffusion minimum inhibitory concentrations (MICs) and by drug gradient plate analysis. MIC and gradient plate experiments revealed diclofenac to significantly increase susceptibility of *S. aureus *to three fluoroquinolone antibiotics in a concentration- and strain-dependent manner. For example, addition of 32 μg/ml diclofenac reduced MICs for ciprofloxacin and norfloxacin in all strains (Table 2) (P < 0.05). MICs were reduced 2-fold in strains SH1000, COL, BB255 and SA1199A, and were reduced by 4- and 8-fold in WBG8287 and WGB9312, respectively. Increasing diclofenac to 64 μg/ml further reduced ciprofloxacin MICs only for SH1000, but had no further impact on norfloxacin MICs. Interestingly, 32 μg/ml diclofenac did not alter ofloxacin MICs for strains SH1000 and COL (MIC = 1 μg/ml) or for BB255 and WGB8287 (MIC = 0.5 μg/ml), but did decrease MICs for strains SA1199B and WGB9312 (P < 0.05) (Table 2). Increasing diclofenac to 64 μg/ml further decreased ofloxacin MICs for SA1199B, but not for WGB9312. Gradient plate analysis for fluoroquinolones supported MIC data, where growth into plates containing 32 μg/ml diclofenac was significantly reduced for SH1000 by 2.8-fold (ciprofloxacin) and 26-fold (norfloxacin) and for COL by 1.5-fold (ciprofloxacin) and 2.2-fold (norfloxacin), but not for ofloxacin for either strain (P < 0.05) (data not shown). Addition of 32 μg/ml and 64 μg/ml diclofenac did not significantly alter MICs for the protein synthesis inhibitors chloramphenicol or tetracycline.

**Table 2:** Effect of diclofenac on antibiotic susceptibility of COL, SH1000 and Dc^RS ^mutant derivatives

				MIC^a ^(μg/ml)		
		
Antibiotic	Strain	Control	Dc^b ^(32 μg/ml)	FI/FD^c^	Dc (64 μg/ml)	FI/FD
Ciprofloxacin	SH1000	0.5	0.25	-2	0.125	-4
	SC1-SC3^d^	0.5	0.25	-2	0.125	-4
	COL	0.5	0.25	-2	0.25	-2
	SC4-SC6^d^	0.5	0.5	0	0.25	-2
	BB255	0.25	0.125	-2	0.125	-2
	WGB8287	0.5	0.125	-4	0.125	-4
	SA1199B	8	4	-2	4	-2
	WBG9312	32	4	-8	4	-8
Norfloxacin	All^e^	0.125	0.0625	-2	0.0625	-2
Ofloxacin	SA1199B	2	1	-2	0.5	-4
	WBG9312	8	4	-2	4	-2
Oxacillin	SH1000	0.25	0.25	0	0.5	2
	SC1-3	0.25	0.5	2	0.5	2
	COL	>256	>256	ND	>256	ND
	SC4-6	>256	>256	ND	>256	ND
	BB255	0.25	0.25	0	0.25	0
	WGB8287	32	64	2	128	4
	SA1199B	0.13	0.25	2	0.5	4
	WBG9312	2	8	4	16	8

^a ^Minimum inhibitory concentration (MIC).

^b ^Diclofenac (Dc).

^c ^Fold increase (FI) or fold decrease (FD) in MIC and in the presence of Dcl.

^d ^DcRS mutant derivative isolates of SH1000 (SC1 through SC3) all had the same MICs; those of COL (SC4 through SC6) also all had the same MICs.

^e ^All (all strains in the study expressed the same MIC: SH1000, COL, SC1-SC6, BB255, WGB8287, SA1199B, and WBG9312).

Diclofenac was also observed to reduce susceptibility of *S. aureus *to the cell wall-active antibiotics oxacillin and vancomycin in a concentration- and strain-dependent manner. Addition of 32 μg/ml diclofenac did not alter oxacillin MICs for SH1000 or BB255, but increased MICs for methicillin-resistant strains WGB8287, SA1199A and WGB9312 (Table 2). Increasing diclofenac to 64 μg/ml increased oxacillin MICs for SH1000, and further increased MICs for WGB8287 and SA1199A, but not for WGB9312. Diclofenac did not alter MICs for vancomycin, but the addition of 32 μg/ml diclofenac did increase growth into vancomycin (2 μg/ml) gradient plates for strains SH1000 from 20 mm to 32 mm (1.6-fold) and WBG8287 from 21 mm to 31 mm (1.5-fold), but not COL and BB255. Gradient plate analysis is sensitive to small but important changes in resistance which may not be detectable by MIC assays. Collectively, the results reveal diclofenac to increase susceptibility to fluoroquinolone antibiotics, and to decrease susceptibility to antibiotics which target the cell wall. This effect of diclofenac on antibiotic susceptibility is strain-dependent, and is generally amplified as the concentration of diclofenac is increased.

### The effect of selection for mutants expressing reduced susceptibility to diclofenac on resistance to antibiotics, and NSAIDs

**Figure 1:**
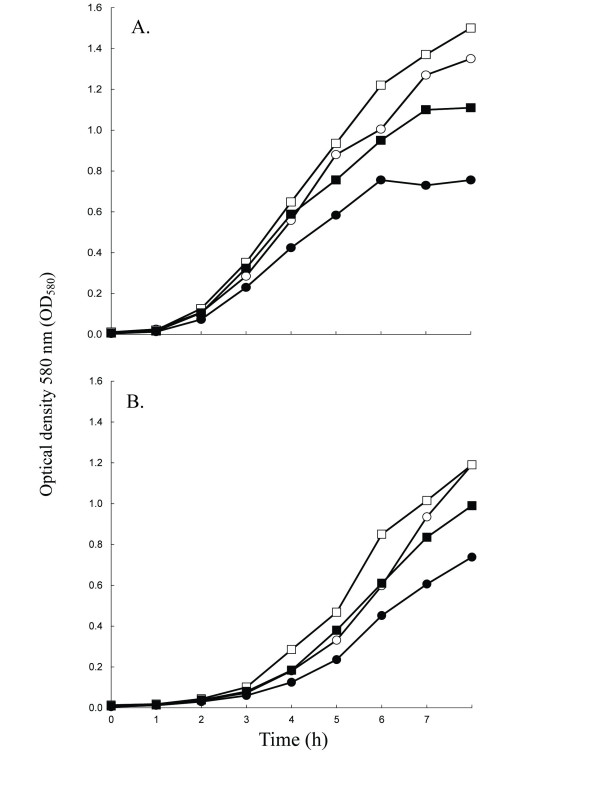
**Growth curve for *S. aureus *strains SH1000 (panel A) and COL (panel B), and their respective diclofenac reduced-susceptibility (Dc^RS^) mutant strains**. Cultures of WT (circles) and Dc^RS ^mutants (squares) were grown in TSB with (filled plots) or without (empty plots) 80 μg/ml diclofenac. The mean optical density is plotted as a function of time for three independent cultures and varied by less than 5%.

To further understand the mechanism by which diclofenac alters resistance, mutants expressing reduced susceptibility to diclofenac (Dc^RS^) were selected by plating overnight MHB cultures (>10^9 ^CFU/ml) on 1X MIC (500 μg/ml) diclofenac gradients followed by incubation (24 h). Dc^RS ^mutants of both SH1000 and COL were isolated from tightly-grouped colonies about 2/3 into the diclofenac gradient. For each strain, three Dc^RS ^mutants were selected and passaged several times on TSA in the absence of diclofenac. For Dc^RS ^mutants (SC1-SC6), diclofenac MICs in MHB increased 4-fold to 2000 μg/ml, and growth of Dc^RS ^mutant SC4 was more vigorous than COL in TSB containing 80 μg/ml diclofenac (Figure 1). Interestingly, SC4 also grew more vigorously in the absence of diclofenac relative to COL (Figure 1).

The Dc^RS ^mutants of COL and SH1000 did not demonstrate altered MICs for the antibiotics included in this study (Table 2). In addition, fluoroquinolone MICs in the presence of 32- and 64- μg/ml diclofenac did not differ between SH1000, COL and their respective Dc^RS ^mutants. Mutation to Dc^RS ^did however alter MICs in the presence of diclofenac for Oxa when compared to SH1000 and COL (Table 2). For example, Oxa MICs increased for Dc^RS ^mutants of SH1000 at 32 μg/ml diclofenac but not at 64 μg/ml, whereas the reverse was true for SH1000. In addition to conferring reduced susceptibility to diclofenac, mutation to Dc^RS ^significantly reduced susceptibility to the NSAID ibuprofen when compared to parent strains (P < 0.05), but did not alter susceptibility to the remaining NSAIDs, or to the salicylate analog, benzoate (Table 3).

**Table 3:** Susceptibility of WT and diclofenac reduced susceptibility (Dc^RS^) mutants to NSAIDs

		Drug gradient plates (mg/ml)^a^				
	
Strain	Ace (0→9)	Asa (0→3.6)	Ben (0→14.4)	Dc (0→0.5)	Ibu (0→4)	Sal (0→8)
SH1000	51 ± 4.2b	24 ± 1.0	54 ± 3.2	13 ± 1.5	0	24 ± 2.1
SC-1	51 ± 3.5	25 ± 0.6	52 ± 3.2	35 ± 5.4*	28 ± 2.3*	27 ± 0.6
COL	35 ± 1.2	22 ± 0.6	39 ± 3.2	23 ± 5.8	12 ± 1.5	31 ± 1.2
SC-4	35 ± 0.6	21 ± 1.5	31 ± 1.5	35 ± 3.6*	21 ± 0*	30 ± 1.2

^a ^Gradient plate technique; drug gradients prepared for acetaminophen (Ace), acetyl salicylic acid (Asa), benzoate (Ben), diclofenac (Dc), ibuprophen (Ibu), and sodium salicylate (Sal); concentration gradient provided in parentheses.

^b ^Average growth into NSAID gradients and standard deviation provided in mm.

* Denotes statistically significant difference between WT and Dc^RS ^by Student's t-test (P < 0.05).

### Effect of diclofenac on ciprofloxacin accumulation

It has been shown previously that the reduced susceptibility of *S. aureus *to ciprofloxacin and ethidium bromide in the presence of salicylate correlates with reductions in the accumulation of these antimicrobials [10]. It was thus hypothesized that increased susceptibility of *S. aureus *grown with diclofenac may result from increased ciprofloxacin accumulation. To test this, accumulation of ciprofloxacin in strain BB255 grown with and without diclofenac was measured fluorometrically. Surprisingly, growth with 32 μg/ml diclofenac resulted in a 29% reduction in ciprofloxacin from 188 ± 57 to 133 ± 19 ng/mg cells (P = 0.01, N = 6). Thus, salicylate and diclofenac both reduce intracellular ciprofloxacin levels, but have opposite effects on resistance to ciprofloxacin: salicylate reduces susceptibility to ciprofloxacin [12], whereas diclofenac increases susceptibility.

## Discussion

Diclofenac has been described as a non-antibiotic broad spectrum antibacterial, which can act in synergy with antibiotics to decrease bacterial cell counts. Support for the latter claim comes from studies showing reductions in MICs and in CFU/ml recovered from infected animals when diclofenac is administered in combination with the protein synthesis-inhibiting aminoglycosides streptomycin and gentamycin, and with the cell wall-active cephalosporins cefotiam and ceftriaxone [25,26,69-71]. For *S. aureus*, only reductions in streptomycin MICs have been reported [17]. How diclofenac is influencing the susceptibility of bacteria to antibiotics is unknown.

In the present study, growth with diclofenac significantly altered the susceptibility of lab and clinical *S. aureus *strains to five of seven antibiotics not previously tested. The study adds the fluoroquinolones ciprofloxacin, ofloxacin and norfloxacin to the list of antibiotics which significantly reduce MICs in the presence of diclofenac. Furthermore, this is the first study to demonstrate that growth with diclofenac can induce phenotypic resistance to antibiotics; namely, to the cell wall-active drugs oxacillin and vancomycin. As anticipated, microarray analysis of *S. aureus *strain COL grown with diclofenac revealed alterations in genes associated with regulation of antimicrobial resistance, and drug efflux. It is thus believed that diclofenac modifies intrinsic mechanisms of phenotypic antimicrobial resistance in *S. aureus*. Similar observations have been made for salicylate and other NSAIDs [7], suggesting that the mechanism by which these drugs influence resistance are at least partially allied. For salicylate, this includes alterations in efflux and a PMF-independent drug permeability barrier, as well as the involvement of MarR-family regulators such as SarA and MgrA [8-10]. In this study, diclofenac was not observed to significantly alter either *sarA *or *mgrA*, but did however strongly down-regulate drug efflux systems encoded by *mepRAB *and the *emrAB*-like operon SACOL2347-2348. Both MepRAB and EmrRAB are important for intrinsic resistance to fluoroquinolones, and *emrRAB *is inducible by salicylate [38,39,45]. It was thus suspected that reduced expression of these efflux systems, leading to intracellular accumulation of antibiotic, might explain the increased susceptibility to fluoroquinolones when grown with diclofenac (Table 2). Instead, diclofenac was observed to reduce intracellular ciprofloxacin levels similar to salicylate (29% for diclofenac, vs. 19% for salicylate) [10]. Importantly, salicylate-inducible resistance to ciprofloxacin can be conferred independent of active efflux [10]. Thus, changes in ciprofloxacin accumulation in the presence of diclofenac, and perhaps salicylate, may not be the direct cause of altered susceptibility to ciprofloxacin and other fluoroquinolones. It is important to note that strain BB255, used in ciprofloxacin accumulation assays, is a *rsbU *derivative, and thus is reduced in σ^B ^activation in response to stress [53,72]. This is perhaps significant, as an intact *sigB *(encoding σ^B^) has been shown to be involved in intrinsic and salicylate-inducible resistance to antimicrobials [9,73], and the expression of *sigB *is up-regulated by salicylate [9], and by diclofenac (Additional File 2). Perhaps more importantly, RsbU has been reported to control the NorA drug efflux pump through MgrA [74]. It is therefore plausible that changes in strain BB255 which confer intrinsic resistance to fluoroquinolones differ mechanistically from those observed in *rsbU*+ strains. In support of this, ciprofloxacin MICs for BB255 were less than all other strains in the study, and reductions in ciprofloxacin MICs in the presence of diclofenac were more marked in *rsbU*+ SH1000 and in the other strains studied (Table 2).

Microarrays also revealed that growth in the presence of diclofenac down-regulates a substantial number of genes important for DNA stability and repair. Fluoroquinolone antibiotics interfere with DNA interactions between gyrase (GyrAB) or topo IV (ParCE), leading to breaks in DNA, and inducing global repair systems such as the SOS response [75,76]. An alternative explanation for the increased sensitivity of *S. aureus *grown with diclofenac to fluoroquinolones may therefore include a reduced ability for repair/turnover of damaged DNA leading to cell death. Interestingly, salicylate has also been shown to alter the expression of DNA biosynthesis/stability genes including *parE *in *S. aureus *[8], and the *pyr *genes in *Bacillus subtilis *[77], and to increase the frequency at which mutation to heritable antibiotic resistance occurs in *S. aureus *for both ciprofloxacin, and the steroid protein synthesis inhibitor fusidic acid [11,12]. Whether or not diclofenac can select for an increased frequency of genotypic resistance to antibiotics, and the significance of these expression differences in this, are important unanswered questions.

Diclofenac was observed to reduce susceptibility to the cell wall active antibiotics oxacillin and vancomycin. Oxacillin is a penicillinase-resistant β-lactam, and vancomycin is a glycopeptide antibiotic which targets _D-_alanyl_-D-_alanine residues in the cell wall, interfering with peptidoglycan biosynthesis. Genotypic resistance to these antibiotics is multifactorial, and includes both lateral gene acquisition and mutation(s) [78,79]. No mechanism of inducible phenotypic resistance to these antibiotics has been described. Moreover, salicylates have not been shown to induce phenotypic resistance to cell-wall active antibiotics. Growth in the presence of diclofenac led to the down-regulation of genes encoding the cell-wall associated penicillin-binding proteins PBP2 (*pbpB*) and PBP4 (*pbp4*), which are required for full resistance expression to β-lactams and vancomycin. For example, a mutation in the ORF of *pbp4 *which abrogates PBP4 production has been identified in laboratory strains which express vancomycin resistance [46], and mutations in the regulatory region of *pbp4 *which lead to PBP4 overproduction have been described in methicillin resistant strains [80]. Furthermore, Boyle-Vavra [81] demonstrated *pbpB *expression was up-regulated by both oxacillin and vancomycin. It is thus possible that *pbpB *and *pbp4 *down-regulation induced by diclofenac contributes to reduced susceptibility to these drugs, the mechanism of which is presently unclear.

Mutation of *sigB *in COL, and in a vancomycin-intermediate *S. aureus *(VISA) strain, was shown to significantly reduce oxacillin and vancomcyin MICs, respectively [82]. Moreover, *in vitro *selection of *S. aureus *mutants which express reduced susceptibility to household disinfectants has been shown to increase resistance to both oxacillin and vancomycin in a *sigB*-dependent manner [73,83]. Together, these findings suggest a role for σ^B ^in intrinsic resistance to antimicrobials which target components of the cell envelope. As diclofenac was determined to alter *sigB *expression by microarrays and qRT-PCR (Additional File 2), the increased expression may also be important for increased resistance to diclofenac-inducible oxacillin and vancomycin. Concordant with this, oxacillin MICs and growth into vancomycin gradients in the presence of diclofenac were not altered in *rsbU *strain BB255, but increased for *rsbU*+ strain SH1000 (Table 2 and data not shown).

*S. aureus *mutants which express reduced susceptibility to diclofenac (Dc^RS^) were not shown to differ in susceptibility to antibiotics compared to parent strains SH1000 or COL. Thus, the cellular alterations which occur at sub-MICs of diclofenac and alter antibiotic susceptibility (i.e. 32-64 μg/ml) are mechanistically-distinct from alterations associated with mutations leading to the Dc^RS ^phenotype selected from 1× MIC (500 μg/ml).

Diclofenac has been shown to significantly reduce *S. aureus *counts from rat granulomatous tissue in the absence of antibiotic [16]. This observation might result from host-specific effects (i.e. immune modulation), or bacterial-specific effects, such as inhibition of growth or altered virulence gene expression. In support of the latter, salicylic acid has been shown to repress *sarA *and SarA-inducible virulence genes such as *hla *(α-hemolysin) and *fnbA *(fibronectin-binding protein) in *S. aureus*, through upregulation of *sigB *[15,20,84]. Although diclofenac was also observed to up-regulate *sigB*, there was no attendant change in *sarA, hla *or *fnbA *expression levels. Similarly, up-regulation of *srrAB *did not lead to the down-regulation of SrrAB-repressed virulence genes such as *agr *RNA III, *tsst-1 *or *spa*. Both *sigB *and *srrAB *products contribute to cellular functions other than pathogenesis including stress durability and anaerobic growth.

## Conclusions

In summary, growth of *S. aureus *with subinhibitory concentrations of diclofenac was shown to alter the expression of hundreds of genes, including those associated with resistance to antimicrobials and with virulence. It was further shown that diclofenac increased the susceptibility of *S. aureus *to the fluoroquinolone antibiotics ciprofloxacin, norfloxacin and ofloxacin. These observations support previous studies which show diclofenac to increase susceptibility of *S. aureus *to the aminoglycoside streptomycin, and to reduce growth and survival of bacterial pathogens in animal models. Furthermore, this is the first study to show that diclofenac can also reduce susceptibility (induce phenotypic resistance) to antibiotics. Significant to *S. aureus*, this included the cell wall active drugs oxacillin and vancomycin, the latter of which is critical for the treatment of severe MRSA infections. The results of this study suggest that diclofenac modifies antimicrobial resistance in *S. aureus*, in part, by altering the expression of regulatory and structural genes associated with cell wall biosynthesis/turnover and transport.

## Competing interests

The authors declare that they have no competing interests.

## Authors' contributions

JR, JG and BW conceived and supervised the study, and prepared the manuscript. JD, SCM, AKS, YS, SZ and NH performed experiments for microarrays, antibiotic susceptibility testing, qRT-PCR and ciprofloxacin accumulation assays. VN and ME contributed to the experimentation, design and data analysis of DNA microarrays. All authors have read and approved the final version.

## Supplementary Material

Additional file 1 Primers used for quantitative real-time PCR (qRT-PCR) in this study Click here for file

Additional file 2 Genes up-regulated following diclofenac induction of S. aureus strain Click here for file

Additional file 3 List of genes which encode hypothetical proteins and which were significantly altered in expression in response to diclofenac Click here for file
