# Supplementary material for: Alterations in the transcriptome and antibiotic susceptibility of Staphylococcus aureus grown in the presence of diclofenac
Source: Ann Clin Microbiol Antimicrob. 2011 Jul 21;10:30. doi: 10.1186/1476-0711-10-30 (PMC3158543; doi:10.1186/1476-0711-10-30)
Supplement: Additional file 1 — Primers used for quantitative real-time PCR (qRT-PCR) in this study [file 1476-0711-10-30-S1.DOC]

**Additional File 1 Primers used for quantitative real-time PCR (qRT-PCR) in this study**

|  |  |  |  |  |
| --- | --- | --- | --- | --- |
| **Gene/** |  |  |  | **Reference/** |
| **locus ID^a^** | ***T_a_*^b^** | **Primer name** | **Sequence** | **source** |
| *brnQ3* | 58 | SACOL1443-F | TGCCAGTAAGTGACATGACG | This study |
|  |  | SACOL1443-R | TTTCCGAATGAACCAAATCC | This study |
| *cap5A* | 60 | SACOL0136-F | CAGTTTATGGCGCAAGAGGT | This study |
|  |  | SACOL0136-R | GCGAAGCTATTCGCAATT | This study |
| *mepA* | 58 | SACOL0405-F | GCGTTGGTGCAGGAACTTAT | This study |
|  |  | SACOL0405-R | GCTGCGATTTGATCACTGAA | This study |
| *mepR* | 55 | SACOL0404-F | ACGAACAGGTCCAACTGTCA | This study |
|  |  | SACOL0404-R | CCCAGAGGTAGTCAGCCCTA | This study |
| *nirB* | 55 | SACOL2398-F | GGTACGCAATACACGACACG | This study |
|  |  | SACOL2398-R | TATCAGGCTGCCCAAGAAGT | This study |
| *rrs* | 55 | 16S-F | TCGTGTCGTGAGATGTTG | Riordan *et al* 2006* |
|  |  | 16S-R | CTGCCCTTTGTATTGTCC | Riordan *et al* 2006 |
| *rsbW* | 60 | SACOL2055-F | TGCCCACATTGTTATTTTCT | This study |
|  |  | SACOL2055-R | TTTCGAGAGCTGGTGCTACA | This study |
| *sigB* | 58 | SigB2-1 | TTTCACCTGAGCAAATTAACCA | Riordan *et al* 2006 |
|  |  | SigB2-2 | TCTTCGTGCTGTGATTGTCCTT | Riordan *et al* 2006 |
| *srrA* | 60 | SACOL1535-F | GTTGCCACCTGGATACCATC | This study |
|  |  | SACOL1535-R | TGGCCAAGAGGCTTATGAAC | This study |
| SACOL2347 | 55 | SACOL2347-F | ACGATTGGTTTTGGTGCTTT | This study |
|  |  | SACOL2347-R | CAACATTGGTGATTTCATTCTT | This study |
| SACOL0301 | 60 | SACOL0301-F | TAATGAAAGCGATCTTCGCC | This study |
|  |  | SACOL0301-R | AGGCGAATATGGTAACACCG | This study |
| SACOL2347 | 60 | SACOL2347-F | ACGATTGGTTTTGGTGCTTT | This study |
|  |  | SACOL2347-R | CAACATTGGTGATTTCATTCTT | This study |
|  |  |  |  |  |

***Riordan, J.T., Muthaiyan, A., Van Voories, W. *et al*.** 2006. Response of *Staphylococcus* *aureus* to salicylate challenge. *Journal of Bacteriology*. **189**(1): 220-227.
