# Supplementary material for: Alterations in the transcriptome and antibiotic susceptibility of Staphylococcus aureus grown in the presence of diclofenac
Source: Ann Clin Microbiol Antimicrob. 2011 Jul 21;10:30. doi: 10.1186/1476-0711-10-30 (PMC3158543; doi:10.1186/1476-0711-10-30)
Supplement: Additional file 2 — Genes up-regulated following diclofenac induction of S. aureus strain [file 1476-0711-10-30-S2.PDF]

Additional File 2 Genes up-regulated following diclofenac induction of *S. aureus* strain COL

			Fold Change[b]	
**Locus ID[a]**	**Gene[a]**	**Function[a]**	**Array**	**qRT-PCR**
**Amino acid metabolism**				
SACOL1329	*glnA*	Glutamine-ammonia ligase	6.9	
**Cofactor metabolism**				
SACOL0538	*ipk*	4-diphosphocytidyl-2-C-methyl-D-erythritol kinase	2.5	
SACOL0564	*pdxS*	Pyridoxine biosynthesis protein	2.8	
SACOL1124	*ctaA*	Cytochrome oxidase assembly protein	2.6	
SACOL2105	*glyA*	Serine hydroxymethyltransferase	2.5	
SACOL2396	*sirB*	Uroporphyrin-III C-methyl transferase	17.5	
**Cell envelope**				
SACOL0052		Glycosyl transferase, group 1 family protein	2.1	
SACOL0054		Mur ligase family protein, authentic frameshift	2.7	
SACOL0136	*cap5A*	Capsular polysaccharide synthesis enzyme	20.1	17.9
SACOL0137	*cap5B*	Capsular polysaccharide synthesis enzyme Cap5B	8.3	
SACOL0138	*cap5C*	Capsular polysaccharide biosynthesis protein Cap5C	5.6	
SACOL0142	*cap5G*	UDP-N-acetylglucosamine 2-epimerase Cap5G	7.9	
SACOL0143	*cap5H*	Capsular polysaccharide synthesis enzyme	3.3	
SACOL0247	*lrgA*	Murein hydrolase regulator LrgA	2.1	
SACOL2019		SdrH protein, putative	2.5	
SACOL2578		Glycosyl transferase, group 2 family protein	7.2	
**Cellular processes**				
SACOL0244	*scdA*	Cell wall metabolism protein	4.9	
SACOL0958		General stress protein 13	2.7	
SACOL1759		Universal stress protein family	9.3	
SACOL1871	*epiG*	Epidermin immunity protein F	10.6	
SACOL1872	*epiE*	Epidermin immunity protein F	4.6	
SACOL2173	*asp23*	Alkaline shock protein 23	6.0	
SACOL2379		Similar to general stress protein	6.6	
SACOL2532		Acetyltransferase	4.3	
SACOL2731		Cold shock protein	2.1	
**Central intermediary metabolism**				
SACOL0660	*adh1*	Alcohol dehydrogenase	4.5	
SACOL2145	*glmS*	D-fructose-6-phosphate amidotransferase	4.3	
SACOL2296		Glycerate dehydrogenase	2.1	
SACOL2535		D-lactate dehydrogenase	2.5	
SACOL2576	*crtN*	Dehydrosqualene desaturase	5.5	
SACOL2577	*crtM*	Squalene desaturase	4.7	

			Fold Change[b]	
Locus ID[a]	Gene[a]	Function[a]	Array	qRT-PCR
**DNA metabolism**				
SACOL1315	*hexA*	DNA mismatch repair protein	2.0	
SACOL1374	*lexA*	LexA repressor	2.6	
**Energy metabolism**				
SACOL2395	*narG*	Respiratory nitrate reductase, alpha subunit	12.1	
SACOL2398	*nirB*	Nitrite reductase large subunit	20.4	15.9
**Fatty acid metabolism**				
SACOL0599		UDP-glucose 4-epimerase related protein	3.3	
SACOL2482	*fabG2*	3-oxoacyl-(acyl carrier protein) reductase	8.4	
**Nucleic acid metabolism**				
SACOL0121	*deoD1*	Purine nucleoside phosphorylase	2.8	
SACOL0790.1		Ribonucleoside-diphosphate reductase 2	3.0	
SACOL0791	*nrdI*	Ribonucleotide reductase stimulatory protein	3.8	
SACOL0793	*nrdF*	Ribonucleotide-diphosphate reductase	2.0	
SACOL2128	*pdp*	Pyrimidine-nucleoside phosphorylase	2.2	
SACOL2634	*nrdG*	Anaerobic ribonucleoside-triphosphate reductase	13.6	
SACOL2635	*nrdD*	Anaerobic ribonucleoside triphosphate reductase	8.0	
**Protein fate**				
SACOL1933		ThiJ/PfpI family protein	3.6	
SACOL2007		Peptidase, M20/M25/M40 family	4.2	
SACOL2295		Staphyloxanthin biosynthesis protein	4.0	
SACOL2671		Translocase	2.3	
**Protein synthesis**				
SACOL0009	*serS*	Seryl-tRNA synthetase	2.8	
SACOL0815		Ribosomal subunit interface protein	10.0	
SACOL1798	*trmB*	tRNA (guanine-N(7)-)-methyltransferase	2.2	
SACOL1803		Pseudouridine synthase, family 1	2.6	
**Regulatory functions**				
SACOL0249		Transcriptional regulator, GntR family	2.1	
SACOL0257		Ribose operon repressor, putative	2.3	
SACOL0518	*treR*	Transcriptional regulator, GntR family	2.0	
SACOL0757		Transcription repressor of fructose operon	5.1	
SACOL1002	*spxA*	SpxA transcriptional regulator	2.0	
SACOL1328	*glnR*	Glutamine synthetase repressor	9.2	
SACOL1534	*srrB*	Staphylococcal respiratory response protein	3.1	
SACOL2147		Transcriptional antiterminator, BglG family	4.7	
SACOL2389		Transcriptional regulatory protein DegU	6.7	
**Signal transduction**				
SACOL1535	*srrA*	DNA-binding response regulator SrrA	4.9	6.2
SACOL2390		Sensory box histidine kinase, putative	5.4	

Locus ID[a]	Gene[a]	Function[a]	Fold Change[b]	
			Array	qRT-PCR
**Transcription**				
SACOL0540		Translation initiation inhibitor homolog	3.9	
SACOL2055	*rsbW*	Anti-sigma B factor	2.3	1.8
**Transport proteins**				
SACOL0086		Putative drug transporter	3.0	
SACOL0088		Similar to transport protein	5.0	
SACOL0097	*sirC*	Iron compound ABC transporter, permease protein	4.9	
SACOL0155		Similar to cation-efflux system membrane protein	2.8	
SACOL0175		PTS II ABC component	4.4	
SACOL0217		Similar to nickel ABC transporter nickel-binding protein	2.3	
SACOL0255		Similar to ribose transporter RbsU	2.4	
SACOL0301		Formate/nitrite transporter protein	16.1	25
SACOL0302		Putative amino acid transport system	5.6	
SACOL0305		ABC transporter, permease protein	5.9	
SACOL0306		Similar to ABC transporter (ATP-binding protein)	7.3	
SACOL0516		Sugar-specific PTS transport system, IIBC component	6.9	
SACOL0620	*proP*	Proline/betaine transporter homolog	2.2	
SACOL0679		Monovalent cation/H+ antiporter subunit A	9.4	
SACOL0680		Na+ antiporter	3.3	
SACOL0682		MnhD homolog	4.3	
SACOL0684		Na+ antiporter	6.9	
SACOL0685		Na+/H+ antiporter, MnhF component, putative	4.2	
SACOL0759		PTS transport system, fructose-specific IIABC component	7.6	
SACOL0796		Ferrichrome ABC transporter permease	2.9	
SACOL0831		CofD-family protein	4.6	
SACOL1476		Amino acid permease	3.6	
SACOL1799		ChoK-family protein	2.4	
SACOL2146		PTS system, mannitol-specific IIBC components	2.7	
SACOL2148		PTS system, mannitol specific IIA component	2.5	
SACOL2176		Glycine betaine transporter opuD homolog	4.9	
SACOL2363		L-lactate permease	25.9	
SACOL2376		PTS system, sucrose-specific IIBC components, putative	6.9	
SACOL2382		Putative proton/sodium-glutamate symport protein	2.4	
SACOL2386	*narK*	Nitrite extrusion protein	31.0	
SACOL2401		Similar to NirC protein	2.0	
SACOL2462		ABC transporter, ATP-binding protein	5.6	
SACOL2514		Putative gluconate permease	4.3	
SACOL2721	*nixA*	High-affinity nickel-transport protein	2.1	
**Unclassified/unknown function**				
SACOL0089		67 kDa Myosin-crossreactive streptococcal antigen homolog	12.1	
SACOL0220		Flavohemoprotein	3.1	

Locus ID[a]	Gene[a]	Function[a]	Fold Change[b]	
			Array	qRT-PCR
SACOL0236		2-C-methyl-D-erythritol 4-phosphate cytidylyltransferase	2.7	
SACOL0541	*spoVG*	SpoVG regulatory protein	3.8	
SACOL0671		Hydrolase, alpha/beta hydrolase fold family	4.8	
SACOL0747		Similar to cobalamin synthesis related protein	2.4	
SACOL0872		OsmC/Ohr family protein	6.6	
SACOL0917		NifU domain protein	2.0	
SACOL1071		Chitinase-related protein	2.3	
SACOL1192		S-adenosyl-methyltransferase mraW	2.3	
SACOL1791		FtsK/SpoIIIE family protein	2.0	
SACOL1846		Putative lipoprotein	4.1	
SACOL1941	*yihY*	Ribonuclease BN, putative	5.2	
SACOL2012		Acetyltransferase, GNAT family	2.0	
SACOL2321		Short chain dehydrogenase	4.2	
SACOL2459		Similar to para-nitrobenzyl esterase chain A	4.7	
SACOL2579		Similar to phytoene dehydrogenase	4.8	

Additional File 2 Genes down-regulated following diclofenac induction of *S. aureus* strain COL

			Fold Change[b]	
**Locus ID[a]**	**Gene[a]**	**Function[a]**	**Array**	**qRT-PCR**
**Amino acid metabolism**				
SACOL0515	*gltD*	Glutamate synthase, small subunit	-2.3	
SACOL1505	*aroB*	3-dehydroquinate synthase	-2.1	
SACOL1920		Similar to D-3-phosphoglycerate dehydrogenase	-2.3	
**Cofactor metabolism**				
SACOL0558	*folP*	Dihydropteroate synthase chain A synthetase	-2.4	
SACOL1922	*gsaB*	Glutamate-1-semialdehyde aminotransferase	-2.4	
SACOL2266		Molybdopterin biosynthesis MoeA protein, putative	-2.6	
SACOL2448		2-dehydropantoate 2-reductase	-2.8	
SACOL2560		Hydroxymethylglutaryl-CoA reductase, degradative	-2.6	
SACOL2615	*panB*	3-methyl-2-oxobutanoate hydroxymethyltransferase	-3.1	
SACOL2616		2-dehydropantoate 2-reductase	-2.1	
**Cell envelope**				
SACOL0050	*pls*	Methicillin-resistant surface protein	-2.1	
SACOL0119	*sasD*	Putative surface anchored protein	-2.0	
SACOL0548		Putative polysaccharide biosynthesis protein	-2.4	
SACOL0699	*pbp4*	Penicillin-binding protein 4	-2.3	
SACOL0935	*dltA*	D-alanine--D-alanyl carrier protein ligase	-2.0	
SACOL0936	*dltB*	DltB protein	-2.0	
SACOL1134	*coaD*	Phosphopantheteine adenyltransferase	-2.0	
SACOL1490	*pbp2*	PBP2	-3.0	
SACOL1804		Putative polysaccharide biosynthesis protein	-2.5	
SACOL1892		Putative transporter protein	-2.0	
SACOL2073	*murF*	UDP-N-acetylmuramoylalanyl-D-glutamyl ligase	-2.0	
SACOL2275		Similar to biotin biosynthesis protein	-2.4	
SACOL2446		Similar to dTDP-glucose 4,6-dehydratase	-5.6	
SACOL2676	*sasA*	LPXTG cell wall surface anchor family protein	-2.0	
**Cellular processes**				
SACOL0555		Cell division protein FtsH, putative	-2.0	
SACOL1368	*katA*	Catalase	-2.9	
SACOL1396	*fmtC*	FmtC protein	-5.5	
SACOL1456		Methionine sulfoxide reductase B	-2.2	
SACOL1824	*arsC*	Arsenate reductase	-2.3	
SACOL1964	*camS*	Staphylococcus aureus sex pheromone	-2.4	

Locus ID[a]	Gene[a]	Function[a]	Fold Change[b] Array	Fold Change[b] qRT-PCR
**Central intermediary metabolism**			-2.1	
SACOL0173		Putative thiamine pyrophosphate enzyme	-3.2	
SACOL1365		HAD superfamily hydrolase	-2.0	
SACOL2021		Carbon-nitrogen family hydrolase	-2.1	
SACOL2280	*ureA*	Urease, gamma subunit	-4.9	
SACOL2281	*ureB*	Urease beta subunit	-11.0	
SACOL2282	*ureC*	Urease, alpha subunit	-9.1	
SACOL2283	*ureE*	Urease accessory protein UreE	-6.0	
SACOL2284	*ureF*	Urease accessory protein UreF	-3.4	
SACOL2286	*ureD*	Urease accessory protein UreD	-3.5	
SACOL2367		Probable oxidoreductase	-5.3	
SACOL2488		Similar to oxidoreductase	-4.1	
**DNA metabolism**				
SACOL0534	*tatD*	Deoxyribonuclease, TatD family	-2.3	
SACOL0572	*radA*	DNA repair protein RadA	-2.6	
SACOL1150	*rnhC*	Ribonuclease HIII	-3.5	
SACOL1154		Recombination and DNA strand exchange inhibitor	-2.0	
SACOL1162		Nucleoside triphosphatase	-2.9	
SACOL1267	*topA*	DNA topoisomerase I	-3.2	
SACOL1390	*parC*	DNA topoisomerase IV subunit A	-2.3	
SACOL1489	*recU*	Holiday junction specific endonuclease	-2.8	
SACOL1540	*xerD*	Tyrosine recombinase	-3.7	
SACOL1614	*nfo*	Endonuclease IV	-2.4	
SACOL2141		Site-specific recombinase family protein	-2.1	
SACOL2594		Short chain oxidoreductase	-39.4	
**Fatty acid metabolism**				
SACOL0426		Acetyl-CoA C-acetyltransferase homolog	-2.5	
SACOL0987	*fabH*	3-oxoacyl-(acyl carrier protein) synthase	-2.7	
SACOL1016	*fabI*	Enoyl-(acyl carrier protein) reductase	-3.1	
SACOL1244	*fabD*	Malonyl CoA-acyl carrier protein transacylase	-2.3	
SACOL1245	*fabG1*	3-oxoacyl-(acyl-carrier-protein) reductase	-2.2	
SACOL1322		Similar to lysophospholipase	-2.9	
SACOL1496		Similar to biotin ligase	-2.7	
SACOL1571	*accC*	Acetyl-CoA carboxylase	-3.0	
SACOL1770		Glycerophosphoryl diester phosphodiesterase	-2.2	
**Mobile elements**				
SACOL1854		Transposase	-2.7	

Locus ID[a]	Gene[a]	Function[a]	Fold Change[b]	
			Array	qRT-PCR
**Protein fate**				
SACOL0417		MttB family protein	-2.5	
SACOL0556		Heat-shock protein HSP33 homolog	-2.7	
SACOL1034		Lipoate-protein ligase A family protein	-2.5	
SACOL1281		Membrane-associated zinc metalloprotease	-2.1	
SACOL1555		Similar to tripeptidase	-2.4	
SACOL1588		Putative peptidase	-2.0	
SACOL1636	*dnaJ*	DnaJ protein	-3.5	
SACOL1637	*dnaK*	DnaK protein	-3.6	
SACOL1638	*grpE*	GrpE protein	-3.7	
SACOL1667		Protease	-2.4	
SACOL1693	*yajC*	Preprotein translocase, YajC subunit	-2.0	
SACOL1722		Trigger factor	-2.0	
SACOL1777		Putative protease	-2.1	
SACOL2016	*groEL*	GroEL protein	-2.3	
SACOL2017	*groES*	GroEL protein	-2.3	
SACOL2038		Similar to O-sialoglycoprotein endopeptidase	-2.3	
SACOL2714	*pcp*	Pyrrolidone-carboxylate peptidase	-2.1	
**Protein synthesis**				
SACOL0439	*rpsR*	Ribosomal protein S18	-2.1	
SACOL0562	*lysS*	Lysyl-tRNA synthetase	-2.1	
SACOL0574	*gltX*	Glutamyl-tRNA synthetase	-2.1	
SACOL0773		Glutamine amidotransferase class-I protein	-2.0	
SACOL0818	*prfB*	Peptide chain release factor 2	-2.0	
SACOL1206	*ileS*	Isoleucyl-tRNA synthetase	-2.6	
SACOL1256	*trmD*	tRNA-mehtyltransferase	-2.3	
SACOL1282	*proS*	Prolyl-tRNA synthetase	-2.5	
SACOL1633		Similar to Fe-S oxidoreductase	-2.2	
SACOL1635		Probable methyltransferase	-2.0	
SACOL1694	*tgt*	Queuine tRNA-ribosyltransferase	-3.3	
SACOL1700	*rpmA*	50S ribosomal protein L27	-2.0	
SACOL1725	*rplT*	50S ribosomal protein L20	-2.5	
SACOL1769	*rpsD*	30S ribosomal protein S4	-2.0	
SACOL1960		Aspartyl/glutamyl-tRNA amidotransferase subunit B	-2.3	
SACOL2221	*rpmD*	Ribosomal protein L30p/L7e	-2.0	
SACOL2226	*rpsN2*	Ribosomal protein S14	-2.4	
SACOL2228	*rplX*	50S ribosomal protein L24	-3.2	
SACOL2229	*rplN*	Ribosomal protein L14	-2.2	
SACOL2233	*rpsC*	30S ribosomal protein S3	-2.9	

Locus ID[a]	Gene[a]	Function[a]	Fold Change[b]	
			Array	qRT-PCR
SACOL2234	*rplV*	Ribosomal protein L22	-3.4	
SACOL2236	*rplB*	50S ribosomal protein L2	-2.2	
SACOL2237		50S ribosomal protein L23	-2.0	
SACOL2238	*rplD*	50S ribosomal protein L4	-2.7	
SACOL2239	*rplC*	Ribosomal protein L3	-2.2	
SACOL2240		30S ribosomal protein S10	-2.9	
SACOL2554.1		LrgA family protein	-2.4	
**Purine/pyrimidine metabolism**				
SACOL0018	*purA*	Adenylosuccinate synthase	-3.0	
SACOL1212		Aspartate carbamoyltransferase catalytic subunit	-7.8	
SACOL1213	*pyrC*	Dihydroorotase	-12.8	
SACOL1214	*pyrAA*	Carbamoyl-phosphate synthase small subunit	-12.7	
SACOL1215	*pyrAB*	Carbamoyl-phosphate synthase large subunit	-11.0	
SACOL1216	*pyrF*	Orotidine 5'-phosphate decarboxylase	-13.8	
SACOL1217	*pyrE*	Orotate phosphoribosyltransferase	-14.2	
SACOL1277	*smbA*	Uridylate kinase	-2.1	
SACOL2119	*pyrG*	CTP synthetase	-2.0	
SACOL2606	*pyrD*	Dihydroorotate dehydrogenase	-2.9	
**Regulatory functions**				
SACOL0307	*pfoR*	Perfringolysin O regulatory protein	-2.6	
SACOL0404	*mepR*	MarR family transcriptional regulator	-2.8	-3.2
SACOL0567	*ctsR*	Putative DNA-binding protein	-2.7	
SACOL1107		Transcriptional regulator, Cro/CI family	-2.6	
SACOL1210	*pyrR*	Pyrimidine regulatory protein PyrR	-3.4	
SACOL1639	*hrcA*	Heat-inducible transcriptional repressor	-3.5	
SACOL1822	*arsR*	Arsenical resistance operon repressor	-3.0	
SACOL2256		MarR family transcriptional regulator	-4.7	
SACOL2593		TetR-family transcriptional regulator	-24.2	
SACOL2645		Putative sensor histidine kinase	-2.0	
**Signal transduction**				
SACOL0765	*saeS*	Sensor histidine kinase SaeS	-2.8	
**Transcription**				
SACOL0582	*nusG*	Transcription antitermination protein NusG	-2.2	
SACOL0588	*rpoB*	DNA-directed RNA polymerase beta subunit	-2.0	
SACOL1255	*rimM*	16S rRNA processing protein RimM	-2.1	
SACOL1471		Similar to cell wall enzyme EbsB	-2.8	
SACOL1665	*greA*	Transcription elongation factor GreA	-2.9	

Locus ID[a]	Gene[a]	Function[a]	Fold Change[b]	
			Array	qRT-PCR
**Transport proteins**				
SACOL0405	*mepA*	MATE-family protein	-9.2	-10.5
SACOL0406	*mepB*	Hypothetical protein	-2.8	
SACOL0954	*mnhB*	Na+/H+ antiporter, MnhB component	-2.0	
SACOL0955	*mnhA*	Na+/H+ antiporter subunit	-2.4	
SACOL1013		Mg2+ transporter	-2.1	
SACOL1108	*potA*	Spermidine/putrescine ABC transporter	-3.0	
SACOL1211	*pyrP*	Uracil permease	-9.7	
SACOL1392		Sodium:alanine symporter family protein	-2.0	
SACOL1414		Oligopeptide transporter putative ATPase domain	-2.0	
SACOL1427		ABC transporter, ATP-binding protein	-2.3	
SACOL1443	*brnQ3*	Branched-chain amino acid transport system protein	-2.4	-5.5
SACOL1728		Amino acid permease	-2.6	
SACOL1728		Lysine-specific permease	-2.4	
SACOL1921		Similar to bacterioferritin comigratory protein	-2.2	
SACOL1924		ABC transporter homolog	-3.4	
SACOL1952		Ferritins family protein	-2.0	
SACOL2156		ATP-binding protein, Mrp/Nbp35 family	-2.2	
SACOL2159		Putative drug transporter	-2.0	
SACOL2246		Glucose uptake protein homolog	-3.5	
SACOL2314		Sodium/bile acid symporter family protein	-2.2	
SACOL2319		Na+ antiporter	-2.2	
SACOL2347		EmrB/QacA family drug resistance transporter	-12.8	-18
SACOL2348		EmrA family drug resistance transporter	-40.7	
SACOL2458		Amino acid permease	-2.6	
SACOL2572	*copA*	Copper-transporting ATPase	-2.6	
**Unclassified/unknown function**				
SACOL0046		Metallo-beta-lactamase family protein	-3.7	
SACOL0435		GTP-binding protein	-2.1	
SACOL0602		Hydrolase, haloacid dehalogenase-like family	-2.7	
SACOL0658		Similar to dGTP triphosphohydrolase	-2.5	
SACOL0778		Sulfatase family protein	-2.0	
SACOL0869		Phosphoglycerate mutase family protein	-2.2	
SACOL0959		NADH-dependent flavin oxidoreductase, Oye family	-8.2	
SACOL1268	*gid*	Glucose-inhibited division protein A	-4.6	
SACOL1305		HD/HDIG/KH domain protein	-2.0	
SACOL1351		Cardiolipin synthetase homolog	-3.4	
SACOL1641	*lepA*	GTP-binding protein LepA	-2.4	
SACOL1699	*obg*	Spo0B-associated GTP-binding protein	-2.3	

			Fold Change[b]	
**Locus ID[a]**	**Gene[a]**	**Function[a]**	**Array**	**qRT-PCR**
SACOL1720		GTP-binding protein	-2.3	
SACOL1835		Plant metabolite dehydrogenase homolog	-2.0	
SACOL2400		Acetyltransferase, GNAT family	-3.3	
SACOL2529		Phospholipase/carboxylesterase family protein	-2.3	
SACOL2591		ActVA 4 protein	-26.9	
SACOL2597		Conserevd hypothetical protein	-3.1	

[a] Locus ID, gene, and functional annotation for *S. aureus* strain COL (GenBank CP000046) (NCBI).

[b] Fold change determined as the expression ratio of diclofenac-induced vs. control (untreated) cultures for microarrays and quantitative real-time PCR (qRT-PCR).
