# Supplementary material for: Alterations in the transcriptome and antibiotic susceptibility of Staphylococcus aureus grown in the presence of diclofenac
Source: Ann Clin Microbiol Antimicrob. 2011 Jul 21;10:30. doi: 10.1186/1476-0711-10-30 (PMC3158543; doi:10.1186/1476-0711-10-30)
Supplement: Additional file 3 — List of genes which encode hypothetical proteins and which were significantly altered in expression in response to diclofenac [file 1476-0711-10-30-S3.PDF]

Additional File 3 List of genes which encode hypothetical proteins and which were significantly altered in expression in response to diclofenac

Locus ID	Fold Change
Up-regulated:	
SACOL0081	2.0
SACOL0092	3.8
SACOL0166	7.3
SACOL0238	2.1
SACOL0444	7.5
SACOL0446	6.3
SACOL0456	2.1
SACOL0457	4.7
SACOL0480	2.3
SACOL0507	2.4
SACOL0537	2.8
SACOL0625	2.3
SACOL0686	4.3
SACOL0723	2.3
SACOL0735	2.1
SACOL0738	2.2
SACOL0742	2.1
SACOL0755	5.2
SACOL0787	3.4
SACOL0862	3.0
SACOL0863	2.9
SACOL0867	2.3
SACOL0868	6.9
SACOL0912	8.6
SACOL1020	2.6
SACOL1033	2.8
SACOL1041	3.1
SACOL1090	5.8
SACOL1167	2.3
SACOL1226	3.3
SACOL1366	2.1
SACOL1387	2.1
SACOL1447	2.1
SACOL1532	2.7
SACOL1679	7.7

Locus ID	Fold Change
Up-regulated:	
**SACOL1680**	**7.0**
SACOL1754	4.4
SACOL1788	2.4
SACOL1789	4.1
SACOL1802	2.2
SACOL1847	2.1
SACOL1895	4.0
SACOL1911	2.4
SACOL1956	2.4
SACOL2076	2.4
SACOL2127	2.5
SACOL2132	2.9
SACOL2136	4.5
SACOL2163	3.3
SACOL2168	2.0
SACOL2169	2.2
SACOL2174	7.4
SACOL2175	5.5
SACOL2197	6.2
SACOL2300	3.7
SACOL2371	2.0
SACOL2383	2.9
SACOL2391	4.4
SACOL2404	2.0
SACOL2433	2.9
SACOL2434	3.3
SACOL2461	5.2
SACOL2467	2.5
SACOL2484	3.1
SACOL2491	10.3
SACOL2532	4.3
SACOL2547	5.4
SACOL2557	4.4
SACOL2603	2.7
SACOL2605	22.0

Additional File 3 cont'd

Locus ID	Fold Change
Up-regulated:	
SACOL2621	9.9
SACOL2625	2.7
SACOL2669	3.2
SACOL2681	4.4
SACOL2711	6.7
SACOL2717	4.8
SACOL2720	5.0
SACOL2723	15.9
SACOL2734	3.8
SACOL0073	2.0
SACOL0206	6.0
SACOL0219	4.7
SACOL0632	7.0
SACOL0866	5.1
SACOL1086	5.8
SACOL1171	2.7
SACOL1356	2.9
SACOL1533	2.3
SACOL1603	3.0
SACOL1640	3.1
SACOL2013	2.4
SACOL2175	7.1
SACOL2365	3.4
SACOL2379	6.6
SACOL2401	5.7
SACOL2481	3.7
SACOL2631	3.7

Locus ID	Fold Change
Down-regulated:	
SACOL0161	-2.0
SACOL0281	-2.5
SACOL0285	-2.0
SACOL0293	-3.3
SACOL0419	-2.1
SACOL0445	-3.6
SACOL0466	-2.9
SACOL0467	-2.7
SACOL0511	-2.6
SACOL0633	-2.1
SACOL0659	-2.2
SACOL0703	-2.7
SACOL0711	-2.1
SACOL0727	-2.0
SACOL0767	-2.2
SACOL0807	-2.6
SACOL1035	-2.3
SACOL1059	-3.5
SACOL1190	-2.4
SACOL1350	-3.2
SACOL1373	-2.1
SACOL1376	-2.3
SACOL1394	-2.1
SACOL1486	-2.0
SACOL1502	-2.9
SACOL1503	-3.7
SACOL1557	-2.8
SACOL1634	-2.1
SACOL1706	-2.5
SACOL1811	-2.0
SACOL1885	-2.1
SACOL1998	-2.0
SACOL2133	-2.0
SACOL2241	-2.1
SACOL2343	-2.0

Additional File 3 cont'd

Locus ID	Fold Change
Down-regulated:	
SACOL2489	-2.5
SACOL2549	-3.3
SACOL2551	-4.1
SACOL2592	-20.1
SACOL2607	-2.7
SACOL0051	-2.2
SACOL0466	-3.8
SACOL0601	-2.4
SACOL0879	-3.6
SACOL0933	-2.0
SACOL1488	-2.5
SACOL1529	-3.5
SACOL2595	-6.3
SACOL2734	-4.9
